# Lethal Injection: Let's Be Honest about the Death Penalty

**DOI:** 10.1371/journal.pmed.0040213

**Published:** 2007-06-26

**Authors:** Lawrence Bonchek

I don't favor the death penalty, and I don't participate in executions, but I recognize that honorable people can disagree about the subject, and I don't consider physicians who wish to do so—to assure that death comes quickly and without unusual pain—to be behaving unethically. They could be seen as serving the interests of both the condemned and society.

The conundrum about lethal injection persists only because long-standing American Medical Association guidelines prohibit physicians from carrying out actual executions, or even pronouncing death, though they may certify it—a distinction without a difference. The lack of physician involvement has resulted in execution protocols based on outmoded pharmacologic methods, carried out by inconsistently qualified technicians, with results that are sometimes ineffective and therefore are understandably controversial.

Any qualified anesthesiologist could propose more reliable techniques. It is unreasonable to assert that a condemned person cannot be executed painlessly, when tens of thousands of people are anesthetized every single day for surgery with modern fast-acting anesthetic drugs (propofol, in particular) that are far more suitable than the outmoded execution drug thiopental. Induction of surgical anesthesia does occasionally cause slight injection pain, so how then can it be “cruel and unusual” to use the same drug and method for the initial step in executions? Next, potassium cannot fail to stop the heart instantly and insensibly if given in substantial amounts.

The debate about the death penalty should be conducted about its morality, not about its methods, because the latter is merely a surrogate for serious debate. Opponents of the death penalty (like me) should recognize that it is unwise to criticize methods alone, because improved methods vitiate those arguments [1].
